# Association between MicroRNA-4669 Polymorphism and Ischemic Stroke in a Korean Population

**DOI:** 10.1155/2019/7238319

**Published:** 2019-11-05

**Authors:** Seoung-Jin Hong, Su Kang Kim, Dong Hwan Yun, Jinmann Chon, Hae Jeong Park

**Affiliations:** ^1^Department of Prosthodontics, Kyung Hee University Dental Hospital, Seoul 02447, Republic of Korea; ^2^Department of Biomedical Laboratory Science, Catholic Kwandong University, Gangneung, Republic of Korea; ^3^Department of Physical Medicine and Rehabilitation, Kyung Hee University, Seoul 02447, Republic of Korea; ^4^Department of Pharmacology, School of Medicine, Kyung Hee University, Seoul 02447, Republic of Korea

## Abstract

Recent studies have explored the association between single-nucleotide polymorphisms (SNPs) in microRNAs (miRNAs) and ischemic stroke (IS). In particular, the associations of rs2910164 (miRNA-146A), rs11614913 (miRNA-196A2), and rs3746444 (miRNA-499A) were intensively studied in IS. In this study, we investigated the associations between SNPs in miRNAs and IS including rs2910164, rs11614913, and rs3746444 in a Korean population. For a pilot study, we selected 19 SNPs in pre-miRNA region (including mature miRNA region) and genotyped in 140 IS patients and 240 control subjects using the Fluidigm Dynamic Array. Our pilot study showed a weak association of rs79402775 in miRNA-933 (*p* = 0.044) and a relatively strong association of rs35196866 in miRNA-4669 (*p* = 0.016) with IS. From the pilot study, we selected rs79402775, rs35196866, and rs7202008 (miRNA-2117; *p* = 0.055) as candidate miRNA SNPs on IS and further genotyped these SNPs in 264 IS patients and 455 control subjects using direct sequencing. In addition, we further analyzed the associations of rs2910164, rs11614913, and rs3746444 that have been intensively studied in previous studies. In the further analysis, we found the significant association between rs35196866 and IS (*p* = 0.0014 in additive model and *p* = 0.00015 in dominant model; *p* = 0.00037 in allele frequency analysis). However, the association between rs2910164, rs11614913, rs3746444, rs79402775, and rs7202008 and IS was not shown. These results suggest that miRNA-4669 may be involved in the susceptibility of IS.

## 1. Introduction

Stroke causes ~11% of all deaths worldwide and is the most common cause of death and adult disability [1]. Ischemic stroke (IS) results from thrombotic occlusion of a cerebral artery or its branches and accounts for about 80-85% of all stroke cases [1]. In Republic of Korea, IS is the third most frequent cause of death after cancer and heart diseases in 2017 (Korea National Statistical Office; http://kostat.go.kr/portal/eng/index.action). IS is a complex disease caused by multiple genetic and environmental risk factors [2]. In addition to the conventional risk factors, including hypertension, diabetes mellitus, smoking, hyperlipidemia, and hyperhomocysteinemia, variants of several susceptible genes have been recognized as genetic risk factors, showing the associations with IS, such as ACE, MTHFR, and APOE [3, 4]. However, the identified susceptible genes explained only a small portion of the total risk of IS, and reliable markers are still lacking.

MicroRNAs (miRNAs) are conserved small noncoding RNAs that function as negative gene regulators. Classical miRNAs are produced from transcripts as primary miRNAs (pri-miRNAs) with several hundred nucleotides (nt) and then are processed into hairpin-structured precursor miRNAs (pre-miRNAs) that have approximately ~70 nt by a RNase Drosha. Pre-miRNAs are exported to the cytoplasm and are further processed by the other RNase Dicer to mature miRNAs of ~22 nt [5]. Matured miRNA could bind to the 3′-untranslated region (3′UTR) of target mRNAs, leading to the regulation of gene expressions through translational repression or mRNA degradation [5]. Recognition of the 3′UTR by miRNAs is mediated through hybridization between seed sequences, which are nucleotides 2 to 8 at the 5′ end of the miRNA and complementary sequences within the 3′UTRs of mRNAs [6–8]. Finally, regulation of mRNA expression depends on perfect or nearly perfect base pairing between the miRNAs and their target sites on the 3′UTRs. A single miRNA could bind to hundreds of mRNA targets, and these targets could be implicated in the regulation of various biological and pathological processes [8–10]. In previous studies, miRNA-146A, miRNA-196A2, and miRNA-499A have been involved in regulating thrombosis or inflammation pathways in the circulation system [11–13]. Indeed, miRNA-146A, miRNA-196A2, and miRNA-499A in mature miRNA regions have been shown to regulate tumor necrosis factor-*α*, Annexin A1, and C-reactive protein [12–14]. In addition, miRNA-146A were differently altered in patients with IS in the acute phase and in the subacute phase [15].

The presence of single-nucleotide polymorphisms (SNPs) in miRNAs could affect the maturation process or target selection of miRNAs [16, 17], thus influencing the risk and/or prognosis of diseases. In particular, rs2910164 in miRNA-146A, rs11614913 in miRNA-196A2, and rs3746444 in miRNA-499A have been extensively studied in the inflammatory or vascular diseases as candidate SNPs [11, 12]. Thereby, these SNPs have been also expected to affect the risk and pathogenesis of IS. Indeed, case control studies have explored the associations between these SNPs (rs2910164, rs11614913, and/or rs3746444) and IS [18–22]. However, the results were inconsistent, even among the same ethnic groups. For example, in Han Chinese populations, several studies reported the significant associations of rs2910164 in miRNA-146A with IS [18, 21] or with large-artery atheroscleric IS [19], whereas other studies reported the lack of association [20, 22].

In this study, we performed the replication study on the genetic association between these 3 SNPs (rs2910164 in miRNA-146A, rs11614913 of miRNA-196A2, and rs3746444 in miRNA-499A) and IS. In addition, we investigated the association between SNPs within other miRNAs and IS and, in particular, focused on SNPs within the pre-miRNA region (including mature and seed miRNA region) which could affect the miRNA maturation and/or the binding to target mRNA.

## 2. Materials and Methods

### 2.1. Subjects

Peripheral blood samples of IS patients used in this study were obtained from patients who were treated in the Stroke Center and the emergency room of Kyung Hee University Medical Center (Seoul, Republic of Korea) between October 2007 and April 2011. All patients were confirmed via cranial CT, MRI, angiography, and duplex sonography. Patients with hemorrhagic stroke, trauma, hematoma, brain tumors, and accidental or iatrogenic stroke were not included in this study. Two hundred and sixty-four samples of peripheral blood were obtained from patients with IS. The National Institutes of Health Stroke Survey (NIHSS) was used in order to measure the neurological functional levels of patients. The modified Barthel index (MBI) was used in order to measure the quality of general life activities.  Table 1 shows the demographic and clinical characteristics of IS patients. The control group comprised 455 age-matched healthy volunteers recruited through a general health checkup program. They had no clinical evidences of IS, transient ischemic attack, ischemic heart disease, or any severe diseases. Written informed consent was obtained from all subjects. If a stroke patient was incommunicative, the informed consent was obtained from a close relative or guardian. This study was conducted in accordance with the guidelines of the Helsinki Declaration and approved by the Ethics Review Committee of the Medical Research Institute, School of Medicine, Kyung Hee University.

### 2.2. SNP Selection and Genotyping

We selected 79 SNPs in miRNAs with minor allele frequency of ≥0.1 in East Asian from MiRSNP database (http://bioinfo.life.hust.edu.cn/miRNASNP2/index.php, version 2) and the National Center for Biotechnology Information (NCBI) SNP database (http://www.ncbi.nlm.nih.gov/SNP, dbSNP BUILD 146). Then, we focused on SNPs in the pre-miRNA region. In the pre-miRNA region, we defined the mature miRNA region including seed region as mature miRNA region and other regions of pre-miRNA except mature miRNA as pre-miRNA region. As a result, 19 SNPs (11 SNPs in the pre-miRNA region and 8 SNPs in the mature miRNA region including 3 SNPs in the seed region) were selected (Table 2).

Genomic DNA was extracted from peripheral blood samples using DNA Isolation Kit for blood (Roche, Indianapolis, IN, USA). As a pilot study, genotyping of 19 SNPs was conducted in 140 patients with IS and 240 control subjects using the Fluidigm Dynamic Array (192.24 Dynamic Array) according to the manufacturer's protocol. The Fluidigm Dynamic Array chip were thermal cycled, and the end-point fluorescent values were measured on Biomark™ system. Final sample-wise genotype cells were obtained using Fluidigm SNP Genotyping Analysis software.

And then, based on our pilot study and previous reports, 6 SNPs were selected and genotyped using direct sequencing in 264 patients with IS and 455 control subjects, including all subjects of the pilot study. Polymerase chain reactions (PCRs) were performed using specific primers for each SNPs ([Supplementary-material supplementary-material-1]). Direct sequencing was performed using an ABI PRISM 3730XL analyzer (PE Applied Biosystems, Foster City, CA, USA). Through direct sequencing, 100% concordant results were observed for duplicated genotyping samples.

### 2.3. Statistical Analysis

SNPStats (http://bioinfo.iconcologia.net/index.php) and IBM SPSS 25 software (SPSS Inc., Chicago, IL, USA) were used to analyze genetic data and the HWE. In order to estimate the genotype association, odds ratio (OR) and 95% confidence intervals (CIs) were calculated using logistic regression analyses, adjusting for age and gender. In logistic regression analysis, additive, dominant, and recessive models were used to determine the associations between SNPs and IS. The chi-squared test was also used for comparison of allele frequencies between groups. In a pilot study, a *p* value lowering 0.05 was considered statistically significant. In further analysis, Bonferroni correction was used to counteract multiple testing problem by lowering the significance levels to *p* = 0.05/6 for the 6 SNPs. Power of sample size was calculated using genetic power calculator (http://zzz.bwh.harvard.edu/gpc/). The differences between SNPs and NIHSS score and MBI score were analyzed using IBM SPSS 25. Statistical comparison of the scores between genotypes for each SNP was assessed by Mann–Whitney *U* test. The *p* value of <0.05 was considered statistically significant.

## 3. Results

For a pilot study, 140 patients with IS (65.1 ± 12.5 years; mean ± SD) and 240 control subjects (67.3 ± 17.7 years) were randomly selected from all subjects (Table 1). Nineteen single-nucleotide polymorphisms (SNPs) selected on the pre-miRNA region of miRNAs were genotyped in these subjects using the Fluidigm Dynamic Array (192.24 Dynamic Array; Fluidigm, South San Francisco, CA, USA). On all 19 SNPs, genotype cell rates of >99.0% were obtained. All SNPs were polymorphic, and the genotype distributions of the SNPs were in the Hardy-Weinberg equilibrium (HWE; *p* > 0.01; [Supplementary-material supplementary-material-1]).  Table 2 shows the allele frequencies of 19 SNPs and allelic associations between 19 SNPs and IS. Among the 19 genotyped SNPs, rs35196866 in miRNA-4669 (A>C) was significantly associated with IS [*p* = 0.016, OR = 0.68, and their 95%CI = 0.48‐0.93]. SNP rs79402775 in miRNA-933 (G>A) also revealed a weak association with IS (*p* = 0.044, OR = 1.50, 95%CI = 1.01‐2.22). Moreover, we performed the multiple logistic regression analysis on 19 SNPs ([Supplementary-material supplementary-material-1]). Although the associations of rs79402775 (*p* = 0.019 in additive model and *p* = 0.025 in dominant model), rs35196866 (*p* = 0.045 in additive model and *p* = 0.047 in dominant model), rs12894467 (*p* = 0.03 in recessive model), and rs7207008 with IS (*p* = 0.0039 in recessive model) were detected, the statistical significances were disappeared after adjustment for false discovery rate (FDR) of multiple tests.

Based on the allele frequency analysis of our pilot study (Table 2), we selected rs79402775 in miRNA-933 (*p* = 0.044), rs35196866 in miRNA-4669 (*p* = 0.016), and rs7207008 in miRNA-2117 (*p* = 0.055). And then, we further genotyped rs79402775, rs35196866, and rs7207008 in all subjects recruited for this study (264 patients with IS (65.1 ± 12.5 years) and 455 control subjects (65.0 ± 16.4 years); Table 1) using direct sequencing and analyzed the genetic association with IS. SNP rs2910164 in miRNA-146A (C>G), rs11614913 in miRNA-196A2 (T>C), and rs3746444 in miRNA-499A (A>G), which are intensively examined in previous studies [18–21], were also genotyped in all subjects. The genotype distributions of rs79402775, rs2910164, rs35196866, rs11614913, rs3746444, and rs7207008 in control subjects were in the HWE (*p* > 0.05). Our study had sample power and size that could determine statistical confidence, considering genotype relative risk of more than 1.8-fold on rs79402775; 1.7-fold on rs3746444 and rs35196866; and 1.6-fold on rs2910164, rs11614913, and rs7207008 (sample power > 0.80, *α* = 0.05). As shown in Table 3, we found that rs35196866 in miRNA-4669 was significantly associated with IS in additive (A/C versus C/C versus A/A; *p* = 0.0014, OR = 0.66, 95%CI = 0.52‐0.86) and dominant models (A/C-C/C versus A/A; *p* = 0.00015, OR = 0.57, 95%CI = 0.41‐0.79). The statistical significances remained after the Bonferroni correction (*p* < 0.05/6). The frequency of the genotypes containing the C allele (A/C-C/C) was decreased in IS patients (41.3%), compared to the control subjects (56.0%). Allele frequency analysis also showed that rs35196866 was associated with IS (*p* = 0.00037, OR = 0.64, 95%CI = 0.51‐0.82). The frequency of the C allele was lower in IS patients (24.4%) than that in control subjects (33.4%).

Moreover, rs79402775 in miRNA-933 showed an association with IS in additive (*p* = 0.016, OR = 1.59, 95%CI = 1.10‐2.31) and dominant models (*p* = 0.022, OR = 1.65, 95%CI = 1.08‐2.53). rs7207008 in miRNA-2117 was also associated with IS (*p* = 0.049, OR = 1.35, 95%CI = 1.00‐1.82 in additive model; *p* = 0.0084, OR = 2.32, 95%CI = 1.26‐4.26 in dominant model). However, the statistical significances on these associations did not remain after the Bonferroni correction. We did not find any association between rs2910164, rs11614913, and rs3746444 and IS.

**Figure 1:**
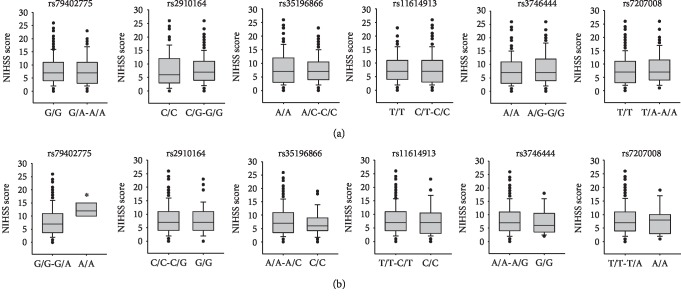
The relationship between miRNA SNPs and National Institute of Health Stroke Scale (NIHSS) score in the dominant (a) and recessive models (b). NIHSS was used in order to measure the neurological functional levels of patients. The difference of NIHSS score between genotypes in the dominant and recessive models was assessed by Mann–Whitney *U* test. ^∗^*p* < 0.05 compared to the reference genotype.

**Figure 2:**
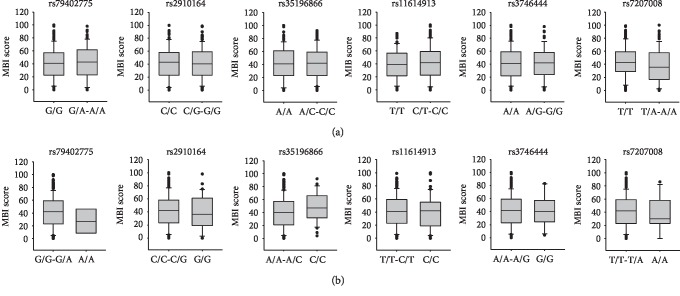
The relationship between miRNA SNPs and modified Barthel index (MBI) score in the dominant (a) and recessive models (b). MBI was used in order to measure the quality of general life activities. The difference of MBI score between genotypes in the dominant and recessive models was assessed by Mann–Whitney *U* test.

Furthermore, we investigated the relationship between SNPs and NIHSS and MBI scores. As shown in Figure 1, in the analysis on NIHSS score, we found a weak relationship between NIHSS score and rs79402775 of miRNA-933 in the recessive model (*p* = 0.0162). The NIHSS score in IS patients with A/A genotype [median (interquartile range) = 12 (11-13.5)] was higher than those with G/G-G/A genotypes [7 (4-11)]. Additionally, the analysis on MBI score showed that in the recessive model of rs35196866 of miRNA-4669, MBI score was increased in IS patients with the C/C genotype [47 (33-65)] compared to that with the A/A-A/C genotype [40 (21.5-56); Figure 2]. However, a significant difference was not detected (*p* = 0.0507).

We also analyzed the association of miRNA SNPs in patient subgroups determined by the presence or absence of hypertension, diabetes, or dyslipidemia; however, no association was observed (data not shown).

**Figure 3:**
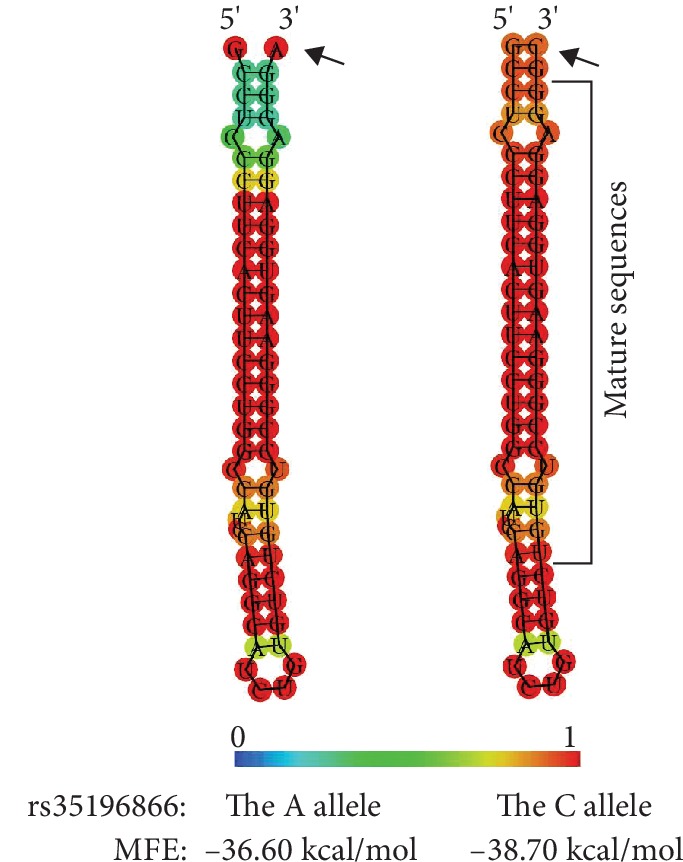
Predicted minimum free energy (MFE) structures of miRNA-4669 by the alleles of rs35196866. MFE structures of miRNA-4669 by the alleles of rs35196866 were predicted using RNAfold web server online software (http://rna.tbi.univie.ac.at/cgi-bin/RNAWebSuite/RNAfold.cgi). The structures are colored by base-pairing probabilities. For unpaired regions, the color denotes the probability of being unpaired. Arrows indicates rs35196866.

rs35196866 is located within the stem-loop sequence of the pre-miRNA-4669 (http://www.mirbase.org/). Thus, it may affect the activity of miRNA-4669 by interfering with the formation of the secondary stem-loop structure and subsequent processing of pre-miRNA-4669 to the mature form. The RNA secondary structure has been predicted by calculation of minimum free energy folding of an RNA sequence. We performed miRNA secondary structure prediction on miRNA-4669 by rs35196866 using the RNAfold web server online software (http://rna.tbi.univie.ac.at/cgi-bin/RNAWebSuite/RNAfold.cgi). As shown in Figure 3, the predicted minimum free energy by the A allele of rs35196866 was −36.6 kcal/mol, whereas the energy by the C allele was −38.7 kcal/mol.

## 4. Discussion

Altered structure of miRNA and binding activity of matured miRNA to target mRNAs have been demonstrated to be implicated in the development of various diseases [23–25]. SNPs in miRNAs could cause these functional alterations of miRNA. The SNPs located in the pre-miRNA region alter the secondary structure of miRNA, influencing the maturation processing of miRNA, whereas the SNPs located in mature miRNA region (including seed regions) directly affect the binding of target mRNA and also the miRNA maturation process [23, 25].

We examined the genetic association between SNPs in miRNAs and IS. In order to detect the possible candidate SNPs for IS, we performed a pilot study on the association with IS, selecting 19 SNPs within the mature miRNA and pre-miRNA regions. In pilot study, we found the associations of rs79402775 in miRNA-933 (*p* = 0.044) and rs35196866 in miRNA-4669 (*p* = 0.016) and the possibility of rs7207008 in miRNA-2117 (*p* = 0.055). Thus, we performed a further study on rs79402775, rs35196866, and rs7207008 using the increased sample size. Interestingly, our further study showed the significant association between rs35196566 and IS. In particular, the frequency of C allele of rs35196866 was decreased in IS patients, compared to control subjects. Moreover, we found that the MBI score was higher in IS patients with the C/C genotype than that with the A/A or A/C genotypes although a statistical significance was not detected. These results indicated that the C allele of rs35196566 was associated with the decreased risk and severity of IS. rs35196566 was located in pre-miRNA region of miRNA-4669, but not in the mature region. Considering previous reports on functions of SNPs in miRNAs [23, 25], rs35196566 may cause structural change in miRNA-4669, affecting the maturation processing of miRNA-4669. Indeed, through the prediction of miRNA secondary structure by calculation of minimum free energy folding of an RNA sequence, we found the predicted minimum free energy by the C allele of rs35196866 was lower than the energy by the A allele. The lower the free energy, the more likely the structure of RNA will form. Thus, in subjects with the C allele, the structure of miRNA-4669 may be stably formed and the maturation process of miRNA-4669 may be facilitated, causing higher level of mature miRNA-4669. Hence, we speculated that the increased level of miRNA-4669 in subjects with the C allele of rs35196566 might be involved in the decreased risk and severity of IS. A recent study reported that the expression of miRNA-4669 was reduced in patients with in-stent restenosis (ISR) in lower extremity arterial occlusive disease although the predicting receiver operating characteristic curve analysis did not show its significance as a diagnostic marker for ISR [26]. Considering this report and our results, a higher level of miRNA-4669 might contribute to the protection of vascular disease such as IS and arterial occlusive disease. Furthermore, a previous study reported the effect of BDNF on regenerative function of endothelial progenitor cells (EPCs), depending on the expression changes of miRNAs [27]. The treatment of BDNF could increase the expression of miRNA-4669 in EPCs [27]. BDNF production was increased on the surface of blood vessels with damaged endothelium, exerting beneficial effects on regenerative function of EPCs [28]. Thereby, miRNA-4669 may also play a role in the recovery of the damaged endothelium after IS when local concentration of BDNF is augmented [29]. Further study on the association between miRNA-4669 and outcome of IS may be needed.

Moreover, we searched the predicted target mRNAs against the miRNA-4669 through the miRDB online database (http://mirdb.org/miRDB/index.html), and 14 predicted targets were detected (e.g., fibroblast growth factor receptor-like 1 (FGFRL1) and neural cell adhesion molecule 1 (NCAM1)) ([Supplementary-material supplementary-material-1]). SNP rs35196866 might induce the alteration in the regulation of all or some of the 14 predicted targets by miRNA-4669, and thus, might be involved in the pathogenesis of IS. For example, a previous study showed that the level of NCAM1 was increased in serums of patients with malignant middle cerebral artery infarction (MMI) compared to those with nonacute cerebral infarction (NACI) [30]. Thus, NCAM1 was suggested as a prediction marker for MMI, being involved in the progression from NACI to MMI. Considering this report and our result, we postulated that the difference of the maturation efficiency of miRNA-4669 between the alleles of rs35196866 might affect the translation and the releasing of NCAM1, leading to the development and/or severity of IS. Future studies about the biological functions of miRNA-4669 and rs35196866 are needed in IS.

We also speculated the associations of rs79402775 and rs7202008 with IS through the pilot study; however, our further analysis showed the lack of association. Nevertheless, we found a weak relationship between NIHSS score and the genotypes of rs79402775 in IS patients. NIHSS score was higher in IS patients with the A/A genotype of rs79402775 than with the G/G or G/A genotypes. A recent study showed that miRNA-933 was significantly decreased in the peripheral circulation of patients with hyperlipidemia and with atherosclerotic cardiovascular diseases (ASCVD) [31]. Thus, authors suggested circulating miRNA-933 as a feasible predictor for ASCVD at the early stage [31]. On the contrary, other study reported that 27-hydroxycholesterol, a cholesterol metabolite, increased the level of miRNA-933 in microvascular endothelial cells along with the elevation of TNF-*α* and IL-6 [32]. Given these reports together with our result, miRNA-933 may play a role in the development and/or severity of vascular diseases although the mechanism has not been established. Further studies about the role of miRNA-933 and the impact of rs79402775 in vascular diseases may be needed.

Furthermore, we analyzed the association between rs2910164, rs11614913, and rs3746444 and IS using the increased sample size. Although our pilot study showed the lack of associations with IS, these SNPs have been intensively studied in IS and could directly affect the interactions between miRNAs and target mRNAs, located in mature miRNA regions of miRNA-146A, miRNA-196A2, and miRNA-499A, respectively. However, like us in our pilot study, any association between these SNPs and IS was not shown in further analysis. On rs11614913, our result was in line with previous studies [18–22]. On rs2910164 and rs3746444, there have been inconsistencies even among previous studies [18–22]. On rs2910164, some studies reported a significant association with IS or with large-artery atheroscleric IS [18, 19, 21], whereas the other study reported the lack of association [20, 22]. Moreover, even in the studies reported, the significant associations, the tendencies of the association were shown differently. Two studies showed the association of the G allele (minor allele) of rs2910164 with an increased risk of IS (Korean and Chinses populations) [18, 21], whereas the other study showed the association with a decreased risk of IS (a Chinese population) [19]. On rs3746444 in miRNA-499, some studies showed the significant associations with IS [20, 22], whereas the other studies reported the lack of association with IS [18, 20]. Further replication studies may be needed to determine the associations of rs79402775 rs2910164, rs11614913, rs3746444, and rs7202008 with IS.

## 5. Conclusions

The present study showed the significant association between rs35196866 in miRNA-4669 and IS in a Korean population. In particular, the C allele of rs35196866 contributed to a decreased risk of IS. This is the first study to describe the possible role of miRNA-4669 in the pathogenesis of IS. However, our study has limitations. We examined only one SNP of miRNA-4669. Hence, it may be that more functionally important or strongly associated SNPs may be missed. In addition, we did not perform the in vitro functional study to elucidate the contribution of the C allele of rs35196866to IS. Moreover, sample power analysis showed that our results had statistical confidence, assuming genotype relative risk more than 1.7-fold on rs35196866. Thus, on rs35196866, an association shown in only dominant model (relative risk = 1.75) would be statistically confident. Our findings need to be validated in further studies with larger sample sizes.

## Figures and Tables

**Table 1:** Clinical characteristics of patients with ischemic stroke (IS).

	IS		Con	
	Pilot (*n* = 140)	Total (*n* = 264)	Pilot (*n* = 240)	Total (*n* = 455)
Male/female (*n*)	74/66	150/114	122/118	210/245
Age (mean age ± SD)	65.1 ± 12.5	65.1 ± 12.5	67.3 ± 17.7	65.0 ± 16.4
Hypertension (absent/present)	42/98	82/182	134/6	437/19
Diabetes mellitus (absent/present)	83/57	165/99	—	—
Dyslipidemia (absent/present)	95/45	165/83	185/55	377/78
CRPS (absent/present)	108/32	210/54		
NIHSS score [median (IQR)]	7.0 (4.0-11.0)	7.0 (4.0-11.0)		
MBI score [median (IQR)]	42.0 (23.3-59.0)	41.0 (23.0-58.5)		

CRPS: complex regional pain syndrome; IQR: interquartile range; MBI: modified Barthel index; NIHSS: National Institute of Health Stroke Scale; SD: standard deviation. Subjects with diabetes mellitus were excluded in the control group.

**Table 2:** Allelic association of single-nucleotide polymorphisms (SNPs) in microRNAs (miRNAs) with ischemic stroke (IS) in a pilot study.

SNP	Gene	Region	Allele	MAF		OR (95% CI)	*p*
				Con (%)	Case (%)		
rs243080	miRNA-4432	Pre-miRNA	A>G	29.7	28.9	0.96 (0.70-1.33)	0.82
rs10175383	miRNA-3679	Pre-miRNA	G>C	33.5	33.2	0.99 (0.72-1.35)	0.94
rs79402775	miRNA-933	Mature	G>A	13.8	19.3	1.50 (1.01-2.22)	**0.044**
rs4674470	miRNA-4268	Pre-miRNA	T>C	20.9	24.6	1.24 (0.87-1.76)	0.24
rs11714172	miRNA-4792	Pre-miRNA	G>T	47.1	50.0	1.12 (0.84-1.51)	0.44
rs12523324	miRNA-4277	Pre-miRNA	A>G	47.5	44.2	0.88 (0.65-1.18)	0.39
rs2910164	miRNA-146A	Seed	C>G	37.9	38.2	1.02 (0.75-1.38)	0.92
rs66683138	miRNA-3622A	Seed	G>A	41.9	35.0	0.78 (0.55-1.01)	0.06
rs35196866	miRNA-4669	Pre-miRNA	A>C	33.3	25.0	0.68 (0.48-0.93)	**0.016**
rs832733	miRNA-4698	Pre-miRNA	C>T	26.3	25.7	0.97 (0.70-1.36)	0.87
rs11614913	miRNA-196A2	Mature	T>C	45.6	46.8	1.05 (0.78-1.41)	0.75
rs2289030	miRNA-492	Pre-miRNA	G>C	18.4	16.8	0.86 (0.58-1.28)	0.47
rs61938575	miRNA-3922	Mature	G>A	17.7	18.3	1.04 (0.71-1.53)	0.83
rs12894467	miRNA-300	Pre-miRNA	T>C	21.5	24.6	1.20 (0.85-1.70)	0.31
rs56103835	miRNA-323B	Pre-miRNA	C>T	26.4	26.6	1.01 (0.73-1.42)	0.94
rs2620381	miRNA-627	Seed	A>C	10.6	13.4	1.30 (0.83-2.05)	0.25
rs8078913	miRNA-4520A	Mature	T>C	30.9	33.5	1.13 (0.82-1.54)	0.47
rs7207008	miRNA-2117	Pre-miRNA	T>A	28.7	35.4	1.36 (0.99-1.87)	0.055
rs3746444	miRNA-499A	Mature	A>G	16.0	15.7	0.98 (0.65-1.46)	0.91

Bold characters represent statistically significant values (*p* < 0.05). “Pre-miRNA” indicates other regions of pre-miRNA except mature miRNA sequences. “Mature” indicates the mature miRNA region excluding seed sequences. “Seed” indicates seed region, nucleotides 2 to 8 at the 5′ end of the mature miRNA. CI: confidence interval; MAF: minor allele frequency; OR: odds ratio.

**Table 3:** Genotypic and allelic association of single-nucleotide polymorphisms (SNPs) in miRNAs with ischemic stroke.

Gene/SNP/location	Model/allele	Genotype	Con	IS	OR (95% CI)	*p*
			*n* (%)	*n* (%)		
miRNA-933	Additive	G/G	335 (73.8)	181 (68.8)	1	
rs79402775		G/A	110 (24.2)	75 (28.5)		
Chr 2		A/A	9 (2.0)	7 (2.7)	1.59 (1.10-2.31)	0.016
	Dominant	G/G	335 (73.8)	181 (68.8)	1	
		G/A-A/A	119 (26.2)	82 (31.2)	1.65 (1.08-2.53)	0.022
	Recessive	G/G-G/A	445 (98.0)	256 (97.3)	1	
		A/A	9 (2.0)	7 (2.7)	2.22 (0.68-7.26)	0.20
	Allele	G	780 (85.9)	437 (83.1)	1	
		A	128 (14.1)	89 (16.9)	1.24 (0.92-1.67)	0.15
miRNA-146A	Additive	C/C	182 (40.4)	110 (41.7)	1	
rs2910164		C/G	208 (46.1)	115 (43.6)		
Chr 5		G/G	61 (13.5)	39 (14.8)	1.04 (0.82-1.31)	0.75
	Dominant	C/C	182 (40.4)	110 (41.7)	1	
		C/G-G/G	269 (59.6)	154 (58.3)	1.01 (0.73-1.40)	0.96
	Recessive	C/C-C/G	390 (86.5)	225 (85.2)	1	
		G/G	61 (13.5)	39 (14.8)	1.14 (0.72-1.80)	0.59
	Allele	C	572 (63.4)	335 (63.4)	1	
		G	330 (36.6)	193 (36.6)	1.00 (0.80-1.25)	0.99
miRNA-4669	Additive	A/A	200 (44.0)	155 (58.7)	1	
rs35196866		A/C	206 (45.3)	89 (33.7)		
Chr 9		C/C	49 (10.8)	20 (7.6)	0.66 (0.52-0.86)	**0.0014**
	Dominant	A/A	200 (44.0)	155 (58.7)	1	
		A/C-C/C	255 (56.0)	109 (41.3)	0.57 (0.41-0.79)	**0.00015**
	Recessive	A/A-A/C	406 (89.2)	244 (92.4)	1	
		C/C	49 (10.8)	20 (7.6)	0.67 (0.38-1.18)	0.16
	Allele	A	606 (66.6)	399 (75.6)	1	
		C	304 (33.4)	129 (24.4)	0.64 (0.51-0.82)	**0.00037**
miRNA-196A2	Additive	T/T	139 (30.6)	85 (32.3)	1	
rs11614913		C/T	209 (45.9)	108 (41.1)		
Chr 12		C/C	107 (23.5)	70 (26.6)	1.03 (0.83-1.28)	0.79
	Dominant	T/T	139 (30.6)	85 (32.3)	1	
		C/T-C/C	316 (69.5)	178 (67.7)	0.91 (0.64-1.29)	0.60
	Recessive	T/T-C/T	348 (76.5)	193 (73.4)	1	
		C/C	107 (23.5)	70 (26.6)	1.22 (0.84-1.77)	0.30
	Allele	T	487 (53.5)	278 (52.9)	1	
		C	423 (46.5)	248 (47.1)	1.03 (0.83-1.27)	0.81
miRNA-499A	Additive	A/A	321 (70.5)	173 (65.8)	1	
rs3746444		A/G	122 (26.8)	76 (28.9)		
Chr 20		G/G	12 (2.6)	14 (5.3)	1.28 (0.96-1.70)	0.10
	Dominant	A/A	321 (70.5)	173 (65.8)	1	
		A/G-G/G	134 (29.4)	90 (34.2)	1.26 (0.89-1.78)	0.19
	Recessive	A/A-A/G	443 (97.4)	249 (94.7)	1	
		G/G	12 (2.6)	14 (5.3)	1.91 (0.84-4.36)	0.12
	Allele	A	764 (84.0)	422 (80.2)	1	
		G	146 (16.0)	104 (19.8)	1.29 (0.98-1.70)	0.07
miRNA-2117	Additive	T/T	222 (49.0)	124 (47.1)	1	
rs7207008		T/A	198 (43.7)	100 (38.0)		
Chr 17		A/A	33 (7.3)	39 (14.8)	1.35 (1.00-1.82)	0.049
	Dominant	T/T	222 (49.0)	124 (47.1)	1	
		T/A-A/A	231 (51.0)	139 (52.9)	1.22 (0.82-1.81)	0.33
	Recessive	T/T-T/A	420 (92.7)	224 (85.2)	1	
		A/A	33 (7.3)	39 (14.8)	2.32 (1.26-4.26)	0.0084
	Allele	T	642 (70.9)	348 (66.2)	1	
		A	264 (29.1)	178 (33.8)	1.24 (0.99-1.57)	0.06

Bold characters represent statistically significant values after Bonferroni correction (*p* < 0.05/6).

## Data Availability

The data used to support the findings of this study are available from the corresponding author upon request.

## Supplementary Materials

Supplementary Materials Table S1: genotypic association of single-nucleotide polymorphisms (SNPs) in miRNAs with ischemic stroke (IS) in a pilot study Table S2: predicted targets for miRNA-4669. Table S3: primer sequences for each SNP.
