# Supplementary material for: Association between MicroRNA-4669 Polymorphism and Ischemic Stroke in a Korean Population
Source: Dis Markers. 2019 Nov 5;2019:7238319. doi: 10.1155/2019/7238319 (PMC6875005; doi:10.1155/2019/7238319)
Supplement: Supplementary Materials — Table S1: genotypic association of single-nucleotide polymorphisms (SNPs) in miRNAs with ischemic stroke (IS) in a pilot study Table S2: predicted targets for miRNA-4669. Table S3: primer sequences for each SNP. [file 7238319.f1.docx]

**Supplemental Materials**

Table S1: Genotypic association of single nucleotide polymorphisms (SNPs) in miRs with ischemic stroke (IS) in a pilot study

| Gene/SNP | Model /Allele | Genotype | Con | IS | HWE-*p* | OR (95% CI) | *p* | **p* |
| --- | --- | --- | --- | --- | --- | --- | --- | --- |
|  |  |  | *n* (%) | *n* (%) |  |  |  |  |
| rs243080 | Additive | A/A | 123 (51.5) | 69 (49.3) |  | 1 |  |  |
| miRNA-4432 |  | A/G | 90 (37.7) | 61 (43.6) |  |  |  |  |
|  |  | G/G | 26 (10.9) | 10 (7.1) | 0.46 | 1.06 (0.69-1.64) | 0.77 | 1 |
|  | Dominant | A/A | 123 (51.5) | 69 (49.3) |  | 1 |  |  |
|  |  | A/G-G/G | 116 (48.5) | 71 (50.7) |  | 0.98 (0.70-1.37) | 0.11 | 1 |
|  | Recessive | A/A-A/G | 213 (89.1) | 130 (92.9) |  | 1 |  |  |
|  |  | G/G | 26 (10.9) | 10 (7.1) |  | 0.92 (0.66-1.28) | 0.63 | 0.63 |
| rs10175383 | Additive | G/G | 111 (46.4) | 59 (42.1) |  |  |  |  |
| miRNA-3679 |  | C/G | 96 (40.2) | 69 (49.3) |  |  |  |  |
|  |  | C/C | 32 (13.4) | 12 (8.6) | 0.73 | 1.07 (0.78-1.48) | 0.67 | 0.94 |
|  | Dominant | G/G | 111 (46.4) | 59 (42.1) |  |  |  |  |
|  |  | C/G-C/C | 128 (53.6) | 81 (57.9) |  | 1.37 (0.88-2.12) | 0.16 | 0.99 |
|  | Recessive | G/G-C/G | 207 (86.6) | 128 (91.4) |  |  |  |  |
|  |  | C/C | 32 (13.4) | 12 (8.6) |  | 0.63 (0.31-1.29) | 0.20 | 0.75 |
| rs79402775 | Additive | G/G | 178 (74.2) | 91 (65.0) |  |  |  |  |
| miRNA-933 |  | A/G | 58 (24.2) | 44 (31.4) |  |  |  |  |
|  |  | A/A | 4 (1.7) | 5 (3.6) | 1 | 1.64 (1.08-2.49) | 0.019 | 0.41 |
|  | Dominant | G/G | 178 (74.2) | 91 (65.0) |  | 1 |  |  |
|  |  | A/G-A/A | 62 (25.8) | 49 (35.0) |  | 1.71 (1.07-2.73) | 0.025 | 0.37 |
|  | Recessive | G/G-A/G | 236 (98.3) | 135 (96.4) |  | 1 |  |  |
|  |  | A/A | 4 (1.7) | 5 (3.6) |  | 2.34 (0.59-9.27) | 0.23 | 0.57 |
| rs4674470 | Additive | T/T | 151 (63.2) | 80 (58.0) |  |  |  |  |
| miRNA-4268 |  | C/T | 76 (31.8) | 48 (34.8) |  |  |  |  |
|  |  | C/C | 12 (5.0) | 10 (7.2) | 0.37 | 1.20 (0.85-1.71) | 0.30 | 0.79 |
|  | Dominant | T/T | 151 (63.2) | 80 (58.0) |  |  |  |  |
|  |  | C/T-C/C | 88 (36.8) | 58 (42.0) |  | 1.24 (0.80-1.93) | 0.33 | 0.86 |
|  | Recessive | T/T-C/T | 227 (95.0) | 128 (92.8) |  |  |  |  |
|  |  | C/C | 12 (5.0) | 10 (7.2) |  | 1.34 (0.55-3.26) | 0.52 | 0.71 |
| rs11714172 | Additive | G/G | 78 (32.5) | 35 (25.4) |  | 1 |  |  |
| miRNA-4792 |  | G/T | 98 (40.8) | 68 (49.3) |  |  |  |  |
|  |  | T/T | 64 (26.7) | 35 (25.4) | 0.023 | 1.09 (0.81-1.45) | 0.57 | 0.89 |
|  | Dominant | G/G | 78 (32.5) | 35 (25.4) |  | 1 |  |  |
|  |  | G/T-T/T | 162 (67.5) | 103 (74.6) |  | 1.45 (0.89-2.34) | 0.13 | 0.69 |
|  | Recessive | G/G-G/T | 176 (73.3) | 103 (74.6) |  | 1 |  |  |
|  |  | T/T | 64 (26.7) | 35 (25.4) |  | 0.86 (0.52-1.41) | 0.55 | 0.99 |
| rs12523324 | Additive | A/A | 63 (26.2) | 44 (31.6) |  | 1 |  |  |
| miRNA-4277 |  | A/G | 126 (52.5) | 67 (48.2) |  |  |  |  |
|  |  | G/G | 51 (21.2) | 28 (20.1) | 0.68 | 0.85 (0.62-1.15) | 0.29 | 0.91 |
|  | Dominant | A/A | 63 (26.2) | 44 (31.6) |  | 1 |  |  |
|  |  | A/G-G/G | 177 (73.8) | 95 (68.3) |  | 0.78 (0.49-1.26) | 0.31 | 0.99 |
|  | Recessive | A/A-A/G | 189 (78.8) | 111 (79.9) |  | 1 |  |  |
|  |  | G/G | 51 (21.2) | 28 (20.1) |  | 0.82 (0.48-1.40) | 0.47 | 0.89 |
| rs2910164 | Additive | C/C | 98 (41.0) | 55 (39.3) |  | 1 |  |  |
| miRNA-146A |  | C/G | 101 (42.3) | 63 (45.0) |  |  |  |  |
|  |  | G/G | 40 (16.7) | 22 (15.7) | 0.13 | 1.05 (0.78-1.42) | 0.75 | 1 |
|  | Dominant | C/C | 98 (41.0) | 55 (39.3) |  | 1 |  |  |
|  |  | C/G-G/G | 141 (59.0) | 85 (60.7) |  | 1.13 (0.73-1.75) | 0.58 | 0.88 |
|  | Recessive | C/C-C/G | 199 (83.3) | 118 (84.3) |  | 1 |  |  |
|  |  | G/G | 40 (16.7) | 22 (15.7) |  | 0.96 (0.54-1.73) | 0.90 | 0.94 |
| rs66683138 | Additive | G/G | 83 (34.6) | 63 (45.0) |  | 1 |  |  |
| miRNA-3622A |  | A/G | 113 (47.1) | 56 (40.0) |  |  |  |  |
|  |  | A/A | 44 (18.3) | 21 (15.0) | 0.20 | 0.77 (0.57-1.04) | 0.088 | 0.34 |
|  | Dominant | G/G | 83 (34.6) | 63 (45.0) |  | 1 |  |  |
|  |  | A/G-A/A | 157 (65.4) | 77 (55.0) |  | 0.69 (0.44-1.06) | 0.091 | 0.42 |
|  | Recessive | G/G-A/G | 196 (81.7) | 119 (85.0) |  | 1 |  |  |
|  |  | A/A | 44 (18.3) | 21 (15.0) |  | 0.73 (0.41-1.31) | 0.29 | 0.70 |
| rs35196866 | Additive | A/A | 106 (44.2) | 79 (56.4) |  | 1 |  |  |
| miRNA-4669 |  | A/C | 108 (45.0) | 52 (37.1) |  |  |  |  |
|  |  | C/C | 26 (10.8) | 9 (6.4) | 1 | 0.71 (0.50-0.99) | 0.044 | 0.31 |
|  | Dominant | A/A | 106 (44.2) | 79 (56.4) |  | 1 |  |  |
|  |  | A/C-C/C | 134 (55.8) | 61 (43.6) |  | 0.65 (0.42-1.00) | 0.047 | 0.40 |
|  | Recessive | A/A-A/C | 214 (89.2) | 131 (93.6) |  | 1 |  |  |
|  |  | C/C | 26 (10.8) | 9 (6.4) |  | 0.65 (0.29-1.46) | 0.28 | 0.97 |
| rs832733 | Additive | C/C | 132 (55.0) | 74 (52.9) |  | 1 |  |  |
| miRNA-4698 |  | C/T | 90 (37.5) | 60 (42.9) |  |  |  |  |
|  |  | T/T | 18 (7.5) | 6 (4.3) | 0.69 | 1.00 (0.70-1.42) | 1 | 1 |
|  | Dominant | C/C | 132 (55.0) | 74 (52.9) |  | 1 |  |  |
|  |  | C/T-T/T | 108 (45.0) | 66 (47.1) |  | 1.17 (0.76-1.80) | 0.48 | 0.93 |
|  | Recessive | C/C-C/T | 222 (92.5) | 134 (95.7) |  | 1 |  |  |
|  |  | T/T | 18 (7.5) | 6 (4.3) |  | 0.50 (0.19-1.32) | 0.14 | 0.68 |
| rs11614913 | Additive | T/T | 73 (30.8) | 48 (34.3) |  | 1 |  |  |
| miRNA-196A2 |  | C/T | 112 (47.3) | 53 (37.9) |  |  |  |  |
|  |  | C/C | 52 (21.9) | 39 (27.9) | 0.22 | 1.05 (0.79-1.41) | 0.73 | 1 |
|  | Dominant | T/T | 73 (30.8) | 48 (34.3) |  | 1 |  |  |
|  |  | C/T-C/C | 164 (69.2) | 92 (65.7) |  | 0.82 (0.51-1.29) | 0.39 | 0.92 |
|  | Recessive | T/T-C/T | 185 (78.1) | 101 (72.1) |  | 1 |  |  |
|  |  | C/C | 52 (21.9) | 39 (27.9) |  | 1.48 (0.90-2.44) | 0.12 | 0.74 |
| rs2289030 | Additive | G/G | 159 (66.5) | 96 (69.6) |  | 1 |  |  |
| miRNA-492 |  | C/G | 72 (30.1) | 39 (28.3) |  |  |  |  |
|  |  | C/C | 8 (3.4) | 3 (2.2) | 1 | 0.89 (0.59-1.35) | 0.58 | 0.98 |
|  | Dominant | G/G | 159 (66.5) | 96 (69.6) |  | 1 |  |  |
|  |  | C/G-C/C | 80 (33.5) | 42 (30.4) |  | 0.88 (0.55-1.39) | 0.58 | 0.86 |
|  | Recessive | G/G-C/G | 231 (96.7) | 135 (97.8) |  | 1 |  |  |
|  |  | C/C | 8 (3.4) | 3 (2.2) |  | 0.87 (0.22-3.49) | 0.85 | 0.82 |
| rs61938575 | Additive | G/G | 163 (67.9) | 92 (66.2) |  | 1 |  |  |
| miRNA-3922 |  | A/G | 69 (28.8) | 43 (30.9) |  |  |  |  |
|  |  | A/A | 8 (3.3) | 4 (2.9) | 1 | 1.01 (0.68-1.50) | 0.98 | 1 |
|  | Dominant | G/G | 163 (67.9) | 92 (66.2) |  | 1 |  |  |
|  |  | A/G-A/A | 77 (32.1) | 47 (33.8) |  | 1.05 (0.66-1.66) | 0.83 | 0.92 |
|  | Recessive | G/G-A/G | 232 (96.7) | 135 (97.1) |  | 1 |  |  |
|  |  | A/A | 8 (3.3) | 4 (2.9) |  | 0.74 (0.21-2.62) | 0.64 | 0.85 |
| rs12894467 | Additive | T/T | 145 (60.4) | 83 (59.3) |  | 1 |  |  |
| miRNA-300 |  | C/T | 87 (36.2) | 45 (32.1) |  |  |  |  |
|  |  | C/C | 8 (3.3) | 12 (8.6) | 0.88 | 1.24 (0.86-1.77) | 0.25 | 0.85 |
|  | Dominant | T/T | 145 (60.4) | 83 (59.3) |  | 1 |  |  |
|  |  | C/T-C/C | 95 (39.6) | 57 (40.7) |  | 1.09 (0.71-1.70) | 0.69 | 0.87 |
|  | Recessive | T/T-C/T | 232 (96.7) | 128 (91.4) |  | 1 |  |  |
|  |  | C/C | 8 (3.3) | 12 (8.6) |  | 2.82 (1.09-7.31) | 0.03 | 0.26 |
| rs56103835 | Additive | C/C | 132 (55.2) | 77 (55.4) |  | 1 |  |  |
| miRNA-323B |  | C/T | 88 (36.8) | 50 (36.0) |  |  |  |  |
|  |  | T/T | 19 (8.0) | 12 (8.6) | 0.23 | 1.03 (0.74-1.45) | 0.84 | 0.99 |
|  | Dominant | C/C | 132 (55.2) | 77 (55.4) |  | 1 |  |  |
|  |  | C/T-T/T | 107 (44.8) | 62 (44.6) |  | 1.02 (0.66-1.57) | 0.94 | 0.98 |
|  | Recessive | C/C-C/T | 220 (92.0) | 127 (91.4) |  | 1 |  |  |
|  |  | T/T | 19 (8.0) | 12 (8.6) |  | 1.14 (0.52-2.49) | 0.74 | 0.82 |
| rs2620381 | Additive | A/A | 190 (79.2) | 103 (74.6) |  | 1 |  |  |
| miRNA-627 |  | A/C | 49 (20.4) | 33 (23.9) |  |  |  |  |
|  |  | C/C | 1 (0.4) | 2 (1.4) | 0.45 | 1.37 (0.85-2.22) | 0.2 | 0.91 |
|  | Dominant | A/A | 190 (79.2) | 103 (74.6) |  | 1 |  |  |
|  |  | A/C-C/C | 50 (20.8) | 35 (25.4) |  | 1.34 (0.80-2.22) | 0.27 | 0.98 |
|  | Recessive | A/A-A/C | 239 (99.6) | 136 (98.5) |  | 1 |  |  |
|  |  | C/C | 1 (0.4) | 2 (1.4) |  | 3.86 (0.32-46.90) | 0.27 | 0.58 |
| rs8078913 | Additive | T/T | 118 (49.6) | 64 (46.0) |  | 1 |  |  |
| miRNA-4520A |  | C/T | 93 (39.1) | 57 (41.0) |  |  |  |  |
|  |  | C/C | 27 (11.3) | 18 (12.9) | 0.12 | 1.12 (0.82-1.53) | 0.47 | 0.83 |
|  | Dominant | T/T | 118 (49.6) | 64 (46.0) |  | 1 |  |  |
|  |  | C/T-C/C | 120 (50.4) | 75 (54.0) |  | 1.15 (0.75-1.77) | 0.52 | 0.88 |
|  | Recessive | T/T-C/T | 211 (88.7) | 121 (87.0) |  | 1 |  |  |
|  |  | C/C | 27 (11.3) | 18 (12.9) |  | 1.20 (0.62-2.30) | 0.59 | 0.87 |
| rs7207008 | Additive | T/T | 116 (48.5) | 62 (44.3) |  | 1 |  |  |
| miRNA-2117 |  | A/T | 109 (45.6) | 57 (40.7) |  |  |  |  |
|  |  | A/A | 14 (5.9) | 21 (15.0) | 0.72 | 1.38 (0.99-1.92) | 0.054 | 0.33 |
|  | Dominant | T/T | 116 (48.5) | 62 (44.3) |  | 1 |  |  |
|  |  | A/T-A/A | 123 (51.5) | 78 (55.7) |  | 1.19 (0.77-1.83) | 0.42 | 0.90 |
|  | Recessive | T/T-A/T | 225 (94.1) | 119 (85.0) |  | 1 |  |  |
|  |  | A/A | 14 (5.9) | 21 (15.0) |  | 2.89 (1.39-5.99) | 0.0039 | 0.058 |
| rs3746444 | Additive | A/A | 168 (70.0) | 100 (71.4) |  | 1 |  |  |
| miRNA-499A |  | A/G | 67 (27.9) | 36 (25.7) |  |  |  |  |
|  |  | G/G | 5 (2.1) | 4 (2.9) | 1 | 0.95 (0.62-1.43) | 0.79 | 1 |
|  | Dominant | A/A | 168 (70.0) | 100 (71.4) |  | 1 |  |  |
|  |  | A/G-G/G | 72 (30.0) | 40 (28.6) |  | 0.92 (0.57-1.48) | 0.73 | 0.86 |
|  | Recessive | A/A-A/G | 235 (97.9) | 136 (97.1) |  | 1 |  |  |
|  |  | G/G | 5 (2.1) | 4 (2.9) |  | 1.11 (0.29-4.29) | 0.88 | 0.92 |

In the logistic regression analysis, to adjust for multiple testing, the Benjamini-Hochberg method was used to control for false discovery rate (FDR). **p* means the *p*-value after adjustment for FDR. A *p*-value lowering 0.05 was considered statistically significant. HWE: Hardy-Weinberg equilibrium; CI: confidence interval; OR: odds ratio.

Table S2: Predicted targets for miR-4669

| Target rank | Target score | Gene symbol | Gene description |
| --- | --- | --- | --- |
| 1 | 91 | TMEM63B | transmembrane protein 63B |
| 2 | 77 | ATCAY | ataxia, cerebellar, Cayman type |
| 3 | 74 | FAM178A | family with sequence similarity 178, member A |
| 4 | 69 | TMEM35 | transmembrane protein 35 |
| 5 | 64 | RIBC1 | RIB43A domain with coiled-coils 1 |
| 6 | 64 | PRDM6 | PR domain containing 6 |
| 7 | 63 | FGFRL1 | fibroblast growth factor receptor-like 1 |
| 8 | 62 | MUC1 | mucin 1, cell surface associated |
| 9 | 58 | RBAK | RB-associated KRAB zinc finger |
| 10 | 52 | ALLC | allantoicase |
| 11 | 52 | B9D2 | B9 protein domain 2 |
| 12 | 51 | NCAM1 | neural cell adhesion molecule 1 |
| 13 | 50 | TCF19 | transcription factor 19 |
| 14 | 50 | KCNQ4 | potassium voltage-gated channel, KQT-like subfamily, member 4 |

Targets for miR-4669 were predicted using the miRDB online database (<http://mirdb.org/miRDB/index.html>).

All the targets have a prediction score in the range of 50-100, with a higher score representing more statistical confidence in the prediction result.

Table S3: Primer sequences for each SNP

| SNPs | Forward/Reverse (5'-3') | Temperature (°C) |
| --- | --- | --- |
| rs79402775 | TCACCAATACACGGTCCTCA | 63 |
|  | TCCAGGGGTGCTTTGTAAAC |  |
| rs2910164 | AGAGATCCACCCACATCAGC | 67 |
|  | GCCTGAGACTCTGCCTTCTG |  |
| rs35196866 | TGCTTGTCCGTGCTTAACTG | 61 |
|  | TACTGGGGCTGTCCTGCTAC |  |
| rs11614913 | AGCAGGAGAGTGCAGGAGAG | 65 |
|  | GAGAGGACGGCATAAAGCAG |  |
| rs3746444 | GTCTTCACTTCCCTGCCAAA | 59 |
|  | GGAGACAGACCCTCCCTCTT |  |
| rs7207008 | ACCCTGTCCCTCAAAACCTT | 63 |
|  | TCTTGGGAGCCAAAAGCTAA |  |

Primers were used for PCR for genotyping of rs79402775, rs2910164, rs35196866, rs11614913, rs3746444, and rs7207008.
